# Comparison of Direct and Indirect cold atmospheric-pressure plasma methods in the B_16_F_10_ melanoma cancer cells treatment

**DOI:** 10.1038/s41598-018-25990-9

**Published:** 2018-05-16

**Authors:** Fariba Saadati, Hamed Mahdikia, Hojjat-Allah Abbaszadeh, Mohammad-Amin Abdollahifar, Maryam Sadat Khoramgah, Babak Shokri

**Affiliations:** 1 Physics Department of Shahid Beheshti University, G.C., P.O. Box, 19839-69411 Tehran, Iran; 2 Laser and Plasma research institute, Shahid Beheshti University, G.C., P.O. Box, 19839-69411 Tehran, Iran; 3 Hearing Disorders Research Center, Loghman Hakim Medical Center & Department of Biology and Anatomical Sciences, School of Medicine, Shahid Beheshti University of Medical Sciences, Tehran, Iran; 4 Department of Biotechnology, School of Advanced Technologies in Medicine, Shahid Beheshti University of Medical Sciences, Tehran, Iran

## Abstract

In this study a novel method was implemented and investigated in order to destroy cancer cells inside the mouse body on a clinical level. In the case of ***in-vitro*** study, MTT assay was employed to discover an effective dose of applied plasma and distinguish the plasma effect in direct and in indirect treatments. Tumor growth was also measured in ***in-vivo*** section so that the effectiveness of direct and indirect treatments could be compared. Furthermore, an investigation was conducted to study the interferences between a conventional method (chemotherapy) and plasma treatment so as to increase the effectiveness of treatment inside the body. Hematoxylin and Eosin, Flow Cytometry, TUNEL and Western Blot assay were used to investigate any cell alteration and the impact of various treatment methods on cancer cell and amount of their apoptosis and protein levels. Radiology and CT scan images were taken to determine the final tumor volume. The results showed a significant cell death and substantial reduction in tumor growth in direct plasma treatment in comparison with indirect plasma treatment. Eventually, dramatic destruction of cancer cells was observed while using of indirect plasma-chemotherapy combination, thus introducing an effective method for deep tissue tumors can be introduced.

## Introduction

Plasma medicine has had a significant growth in recent years^1–3^. Technologies such as plasma surgery to remove lesions was fundamentally based on plasma deadly effects on living systems^4,5^. Nowadays, the effect of cold atmospheric plasma (CAP) on living cells and tissues has led scientists into investigating more on this issue^6–9^. In low temperature plasma, ion temperature is close to room temperature, while electron temperature is in order of a few thousands. High temperature electrons strengthen the plasma through the formation of different states and the direct influence of electron ionization^10–12^. Furthermore, high temperature electron can separate molecular gases like oxygen and nitrogen. These sources produce Reactive Oxygen Species(ROS), Reactive Nitrogen Species (RNS) and other reactive species as well, which is important in biomedicine^13^. Among them, oxygen species such as hydroxyl radical (OH)^14^, atomic oxygen (O_2_)^15^, hydrogen peroxide (H_2_O_2_)^16^, super oxide(O_2_^−^)^16^, ozone (O_3_)^17^ and nitrogen species such as nitric oxide (NO)^17^, nitrogen dioxide (NO_2_)^18^,nitrogen trioxide (NO_3_)^18^, nitrous oxide (N_2_O)^18^, dinitrogen tetroxide (N_2_O_4_)^18^ and also positive ions such as N_2_^+^^19^ are produced by cold plasma^14,20,21^. Iza *et al*. have conducted an investigation on plasma with emphasis on biological applications showing that plasma sources can be cheap and portable. These devices could have various designs having applications such as food sterilization, blood coagulation, skin rejuvenation, wound healing and other skin diseases, dental applications and anti-tumor effects on apoptotic processes^22^.

Over the last few years, cancer has been recognized as a gene mutation disease with various involved genes called oncogenes targeting specific signaling molecules. However, cancer-related mutations and multiple signaling pathways lead to cancer and create complexity^23^.

Conventional treatments haven’t met the satisfaction of scientists and patients due to the complexity of mechanism of this disease and disadvantages such as high cost and its side effects on healthy tissues. Cancer treatment by using cold plasma has drawn attention to itself. Compared to other conventional methods, Cold plasma treatment is cheap and fast and hopefully is a reliable alternative^24–27^.

Reaction of CAP with cancer cells in *in-vivo* and *in-vitro* shows anti-tumor effects^28–33^. Such reaction is resulted from combination of physical and chemical factors. UV photons, heat and electric fields are the physical factors^34,35^. Chemical factors contain tens of active species produced in a gaseous phase by cold plasma^36^. Friedman *et al*. used cold plasma for cancer treatment and showed that high doses of plasma leads to necrosis death and low doses to apoptotic death post treatment^37^. Keidar *et al*. treated Bladder and B16/F10 melanoma cancers *in-vivo* and observed that the tumors with initial size of less than 5 mm disappeared completely; however, larger tumors underwent a reduction in size and maintained their size even after three weeks post treatment^30^.

Transferring plasma into the body is an important and challenging subject especially for deep tumors^25^, while plasma radiation is restricted to the skin and it leads to cell death only in the upper three to five cell layers^26^. Therefore, scientists are looking for a way to transfer plasma inside the body^38–42^. Utsumi *et al*. showed that plasma activated medium reduced tumor size when injected into the mouse body. They also studied the effect of plasma activated medium on Ovary cancer cells both *in-vitro* and *in-vivo*. They noticed 30% reduction of *in-vitro* surviving rate and 69% reduction in tumor cell growth^33^. Keidar *et al*. also showed that the plasma activated medium treatment induced cancer cell death *in-vitro* study^41^. In the plasma activated medium, reactive species are produced in the gas phase such as NO_3_, NO_2_, and H_2_O_2_. These species interact with cell surface and enter the cell through the cell membrane, eventually destroying the mitochondrial networks in cancer cells via Caspase apoptotic pathway^43^. Tanaka *et al*. treated the Glioblastoma tumor cells as well as the normal astrocytes cells with the plasma activated medium. They observed that Glioblastoma cells were perished selectively by this medium. They also showed that extracellular signal-regulated kinase (ERK) and protein kinase B (AKT) signaling pathways were set in a low level by means of the plasma activated medium. According to these results it could be concluded that the plasma activated medium reduces the multiplication and surviving signals^44^. Judee *et al*. also studied the effect of the plasma activated medium on spherical HCT116 multicellular tumor related to Colon cancer and observed DNA damage and suppression of tumor growth^45^. Cheng *et al*. showed that CAP kills cancer cells with minimal damage to normal cells^46^. In fact, high sensitivity of cancer cells to Reactive Oxygen Species (ROS) is due to metabolic differences between cancer cells and normal cells^23^. Cancer cells ROS levels are higher than normal cells condition. Therefore, by CAP exposure, ROS concentration in cancer cells increases so rapidly that it dominates antioxidant defense and causes cell deaths. Since low concentration of ROSs can be tumorigenic, ROS elimination sources must be reduced in cancer cells^47^. Reduction-oxidation reaction (Redox) treatment increases ROS levels in both cancer cells and normal cells. High levels of Antioxidant in normal cells prevent ROS concentrations to reach the threshold of apoptosis. In cancer cells, ROS levels are lower than of normal cells, therefore cancer cells cannot overcome apoptosis^48,49^. In this study, treatment has been done by using direct and indirect plasma exposure *in-vivo* and *in-vitro* states on metastatic melanoma B_16_F_10_ cancer cells to study and understand the effectiveness of plasma direct exposer and plasma activated medium. That plasma to be used inside the body, chemotherapy and indirect plasma were combined.

## Results

### Plasma spectroscopy, characterization

**Figure 1:**
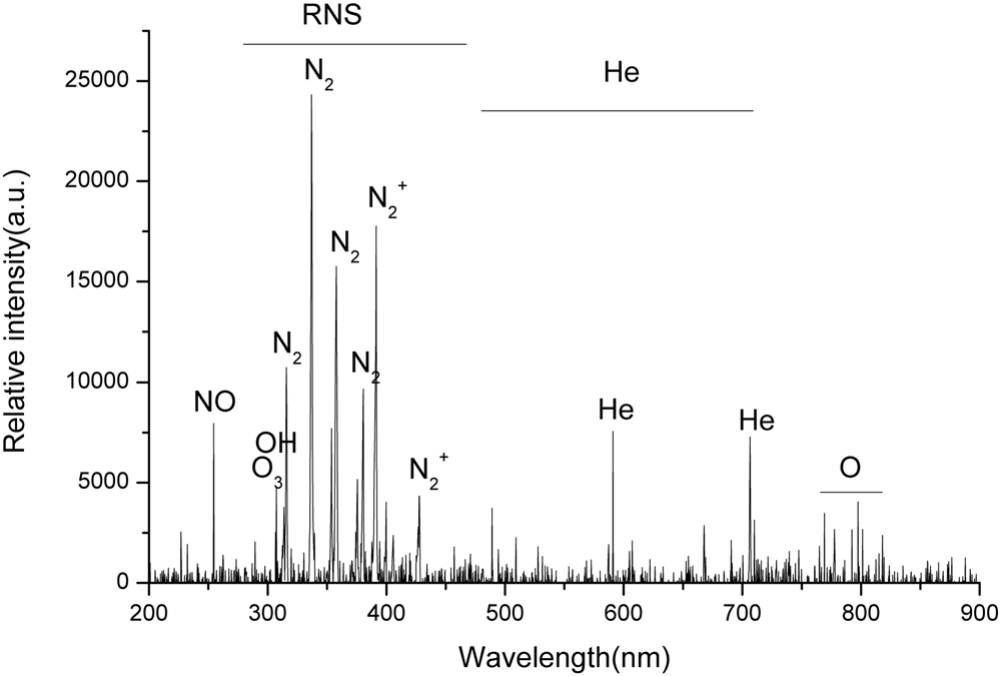
Optical emission spectroscopy of the plasma jet in the range of 200–1000 nm.

Figure 1A represents the results of reactive species intensity measurements, produced by plasma. Different species were determined on the spectrum. ROSs and RNSs containing NO (254 nm), O_3_ (308 nm), OH (310 nm), N_2_ (315 nm–380 nm), N_2_^+^ (391 nm–428 nm) and O (777 nm) have the highest intensity in the spectrum, and also have high importance in plasma medicine and biological applications^15,40,50–52^.

### Cell cultured medium temperature post plasma treatment

**Figure 2:**
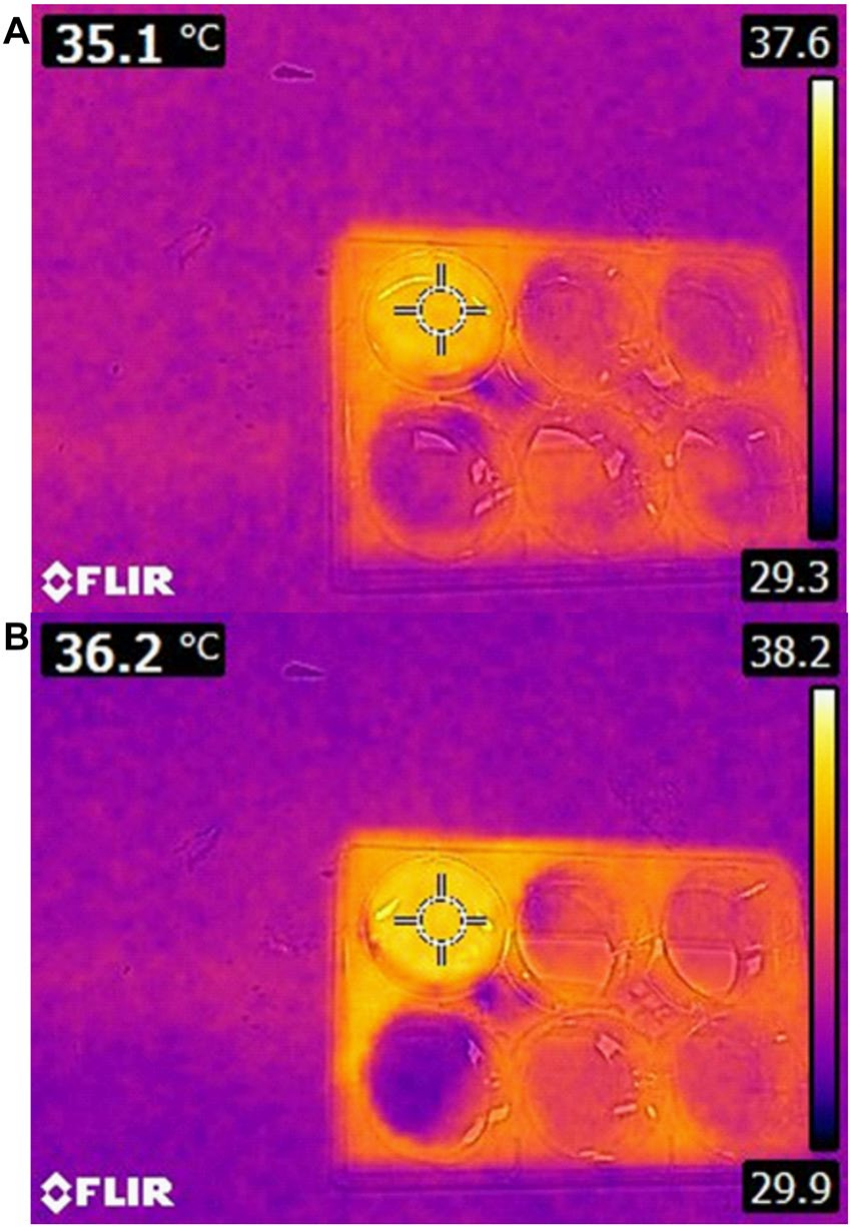
Cell cultured medium (**A**) Before and (**B**) After 6 minutes plasma treatment.

Increasing temperature causes cancer cell death. As mentioned before, the CAP applied for this study is in order of room temperature and has high concentration of ROSs. In this section, to evaluate the thermal damage of CAP, Infra-Red thermometer was implemented. In order to show that this device has no thermal effects, cell cultured medium temperature was measured with Infra-Red thermometer after 6 minutes of plasma treatment and 1 ± 0.1 °*c* temperature increase was observed. This temperature change cannot inflict thermal damages in cells (Fig. 2).

### Mouse skin temperature after plasma treatment

**Figure 3:**
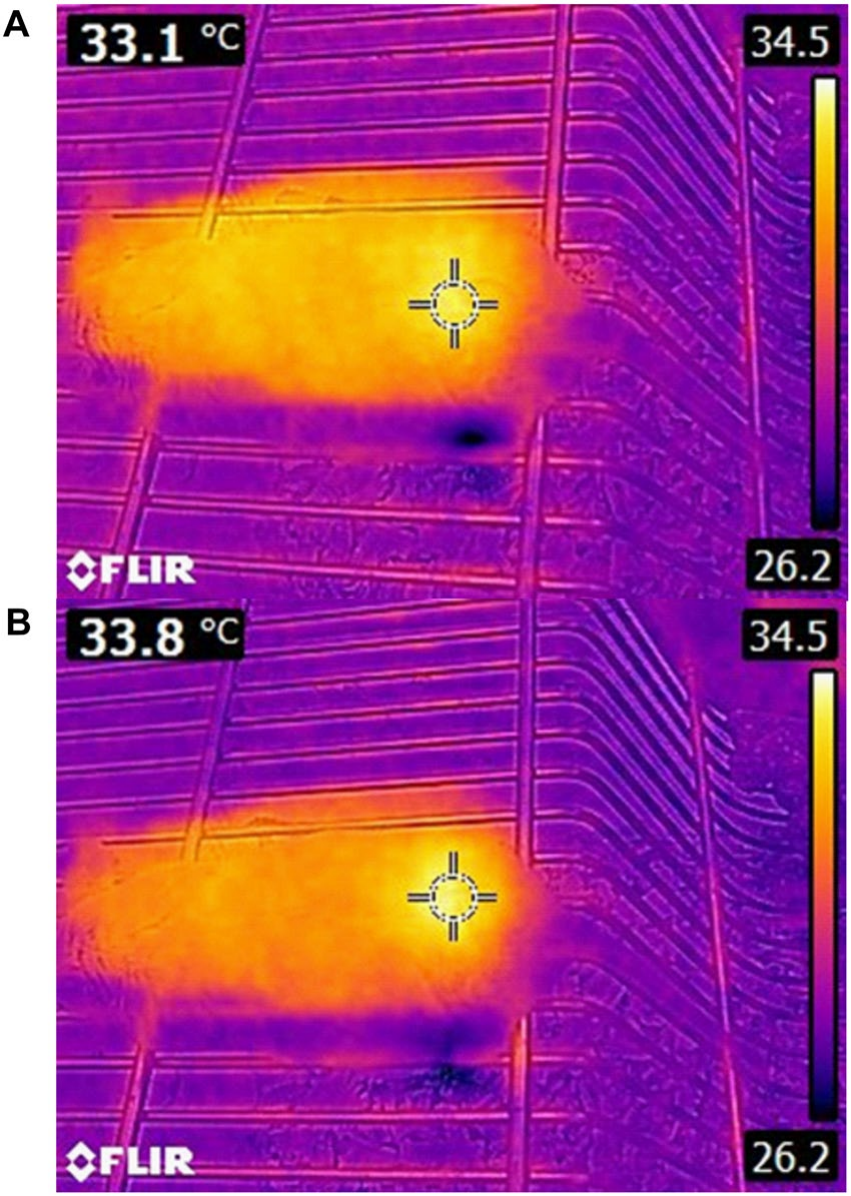
Skin mouse temperature (**A**) Before and, (**B**) After 5 minutes plasma treatment.

Mouse skin temperature was measured by Infra-Red camera in *in-vivo* study and the increase of skin temperature after 6 minutes of plasma exposure was reported to be 1  ±  0.3 °*c*, which cannot cause thermal damage (Fig. 3).

### MTT assay

**Figure 4:**
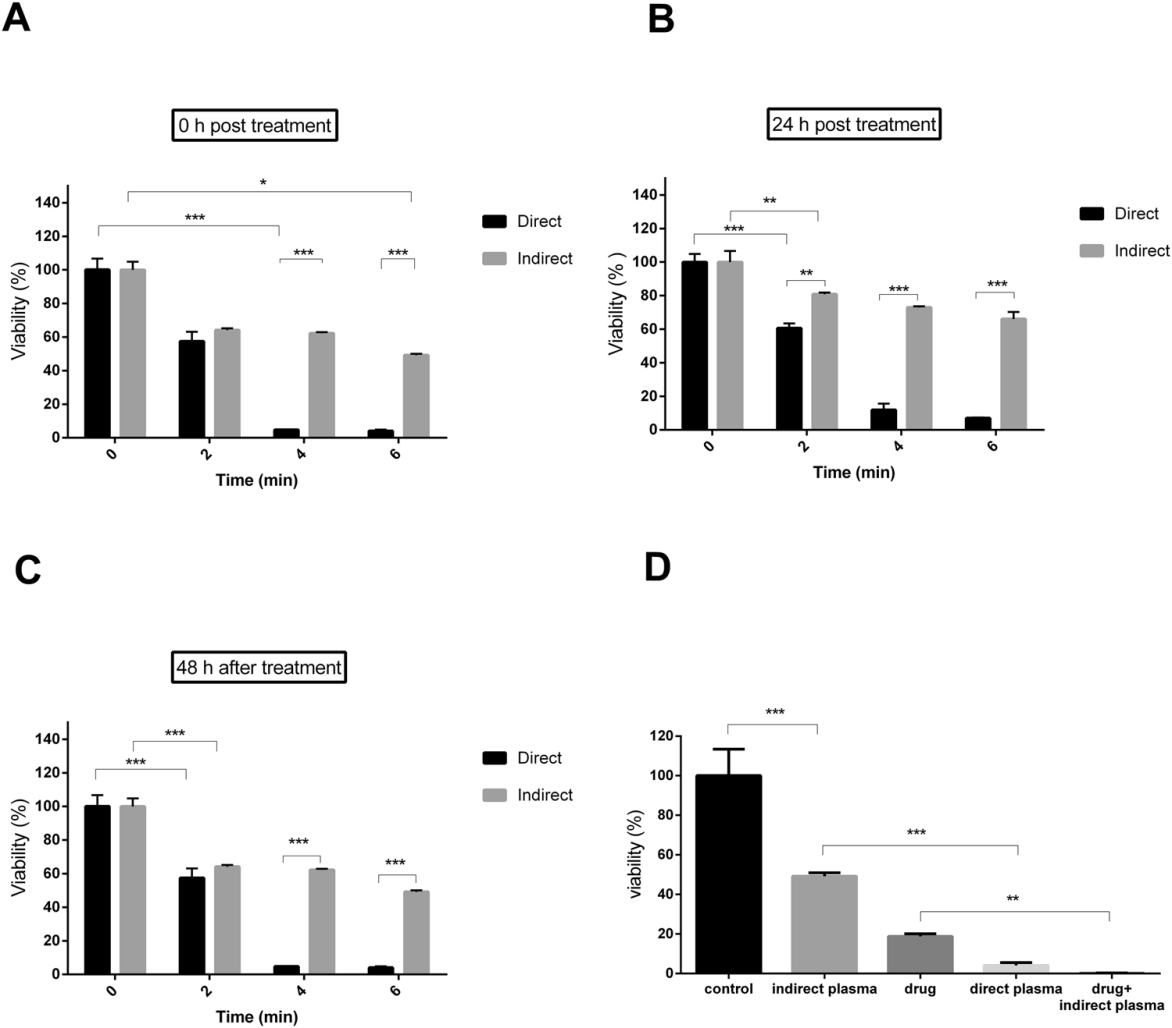
MTT assay (**A**) 0 hour after treatment, (**B**) 24 hour after treatment and (**C**) 48 hour after treatment. (**D**) Comparison of the cell death in diverse groups. (P-values < 0.05 (*), P-values < 0.01 (**) and P-values < 0.001 (***)).

As shown in Fig. 4 the cell death in the direct treatment is more than in the indirect treatment^53^. The best result was for the direct treatment with 45% cell death in 2 minutes exposure time, 94% in 4 minutes and 95% cell death in 6 minutes exposure time based on analysis conducted 48 hours after the treatment. Also, the results for indirect treatment were 38% cell death in 2 minutes, 42% cell death in 4 minutes and 55% cell death in 6 minutes exposure time based on analysis conducted 48 hours after treatment. Eventually, since the best result was shown for 6 minutes and 48 hours after treatment, this condition was selected to be used for comparative study (Fig. 4A–C). Indirect plasma was combined with chemotherapy and as a result the cell viability deducted to 0.19% compared to control group. This amount compared to viability of 4.07% in direct plasma and 18.77% in chemical drug is considerable.

### Flow cytometry assay

**Figure 5:**
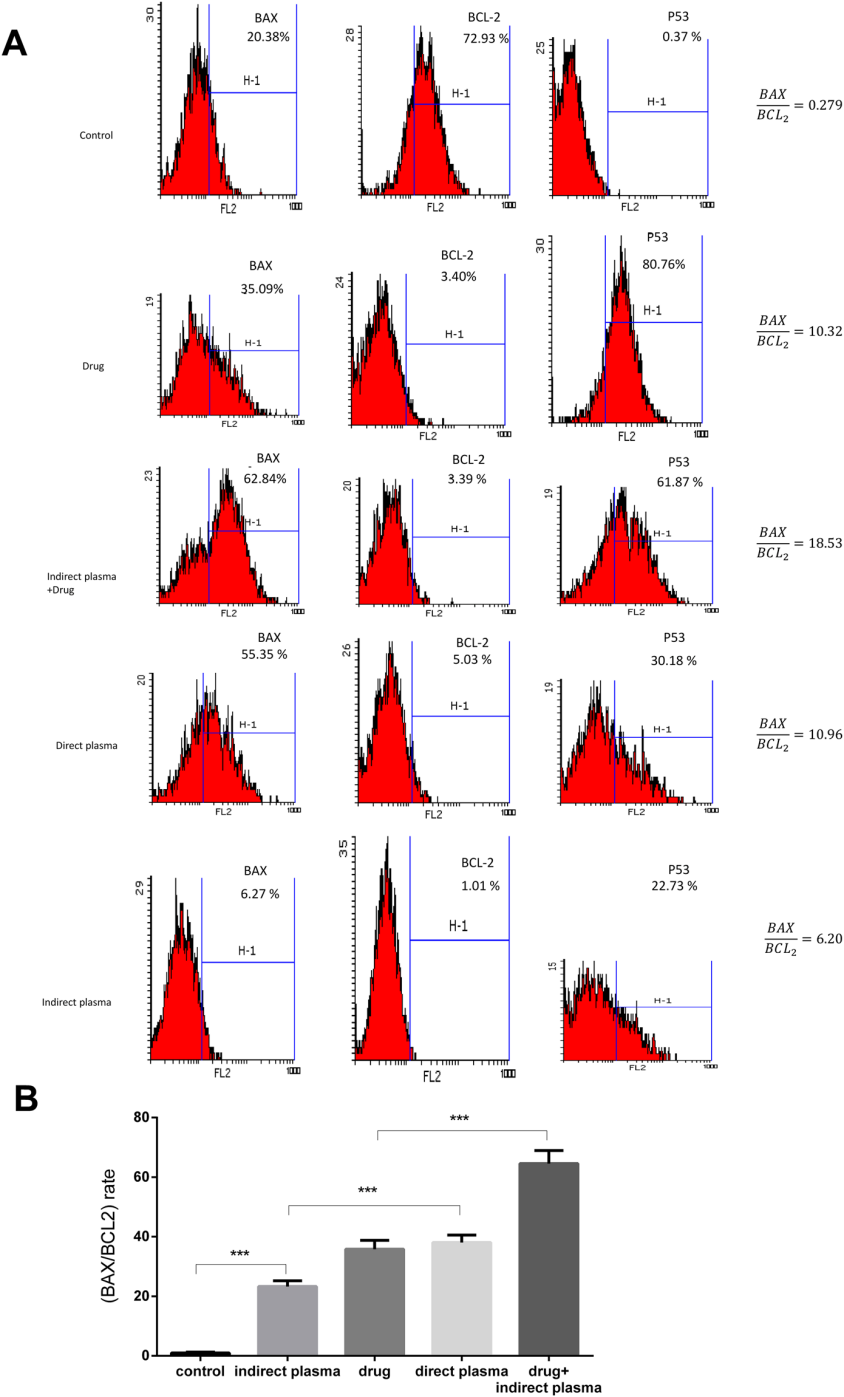
(**A**) Diagram of flow cytometry assay in control and treatment group. (**B**) Rate in untreated, direct and indirect plasma, chemotherapy drug and combination of indirect plasma and chemotherapy drug treatment groups. (P-values < 0.05 (*), P-values < 0.01 (**) and P-values < 0.001 (***)).

Figure 5 represents that apoptosis was induced to the control and treatment groups in B_16_F_10_ melanoma cancer cells based on flow cytometry results. For the first part of the experiment, $\frac{Bax}{Bc{l}_{2}}$ rate for direct treatment is more noticeable than indirect treatment. Actually, $\frac{Bax}{Bc{l}_{2}}$ rate for direct treatment is 10.96, for indirect treatment is 6.20, for chemotherapy drug is 10.32, and for control group is 0.279.

Combinational therapy group of indirect plasma and chemotherapy drug with 18.53 $\frac{Bax}{Bc{l}_{2}}$ rate and 66.4% apoptosis has the best effectiveness compared to the other groups. In addition, apoptosis in direct treatment was 38.07, in indirect treatment was 23.27, and in drug treatment was 35.81. Besides, the expression of p53 was 0.37%, 22.73%, 30.18%, 80.76% and 61.87 in the untreated, indirect plasma, direct plasma, drug, and combined groups, respectively. The results demonstrated that p53 expression of treated groups is significantly different from that of untreated group.

### Tumor size

**Figure 6:**
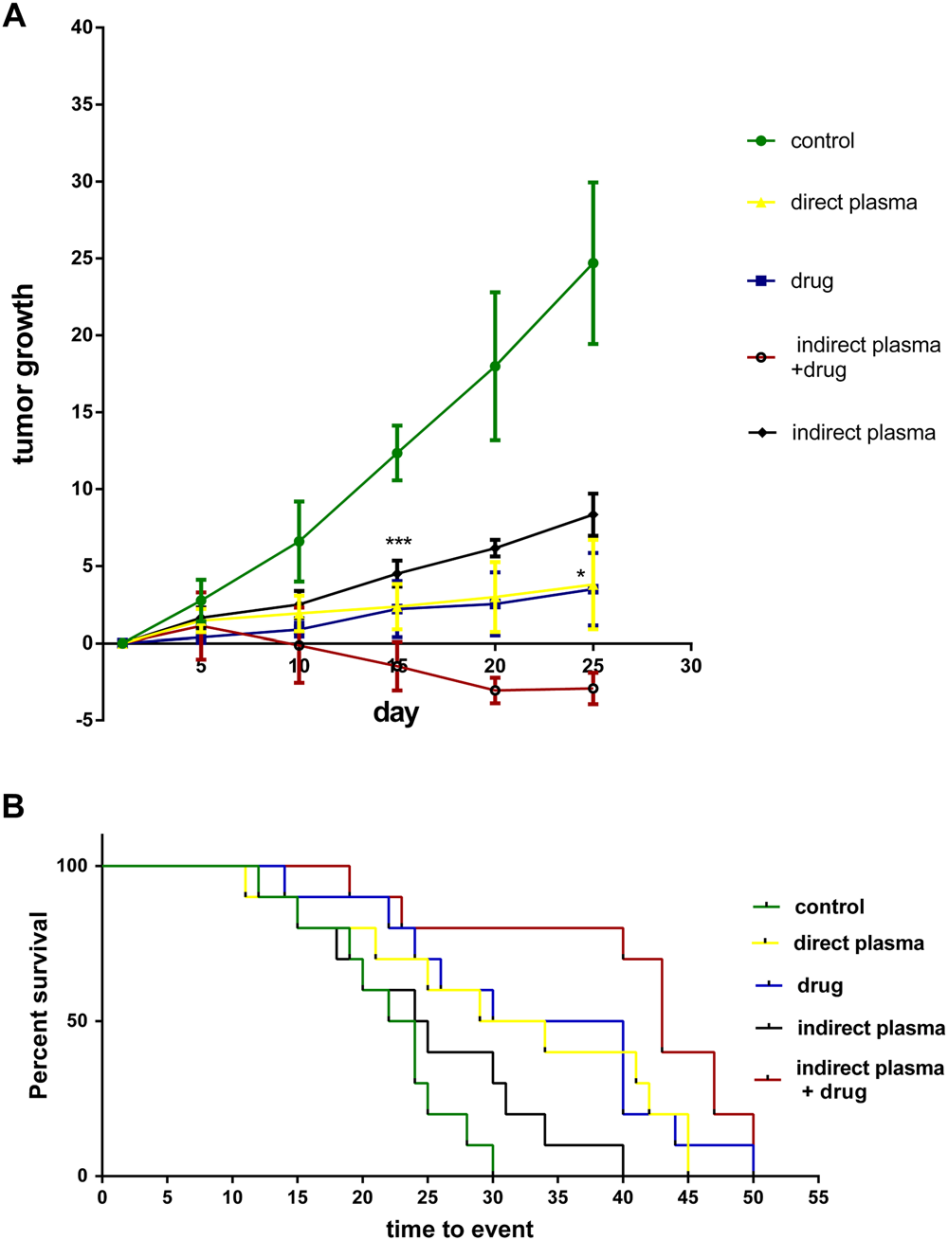
(**A**) Tumor growth measurements during the 25 days after direct and indirect plasma, drug and combinational treatments groups compared to the control group. (**B**) Survival rate of the animals after treatment. (P-values < 0.05 (*), P-values < 0.01 (**) and P-values < 0.001 (***)).

Figure 6 shows mice tumor growth of direct and indirect plasma exposure, chemotherapy drug and indirect plasma combined with chemotherapy drug treatment groups over 25 days of treatment. There was a significant difference between the control and treated groups that is shown in the diagram 6A. Direct plasma has shown a better result than indirect treatment, although combination of indirect treatment with chemotherapy had even showed better results such as negative growth^54^. In other words, with fewer side effects, reduction of tumor size was achieved by this new method. Best results were found for combinational therapy group for which the volume of tumor reached to 2 ± 0.3 mm^3^ in mice having original tumor volume less than 100 mm^3^ after treatment. Furthermore, after the treatment, the survival rate of mice was investigated. Use of CAP treatment in both direct and indirect treatment causes long life span. The average life time of mice in direct treatment group was more than that of indirect ones. The results of combined group demonstrated that 70% of mice survived for 40 days. Moreover, all mice of control group were dead by the 30^th^ day. There were significant differences in survival rate between control and treatment groups as shown in Fig. 6B.

### Hematoxylin and Eosin (H&E) result

**Figure 7:**
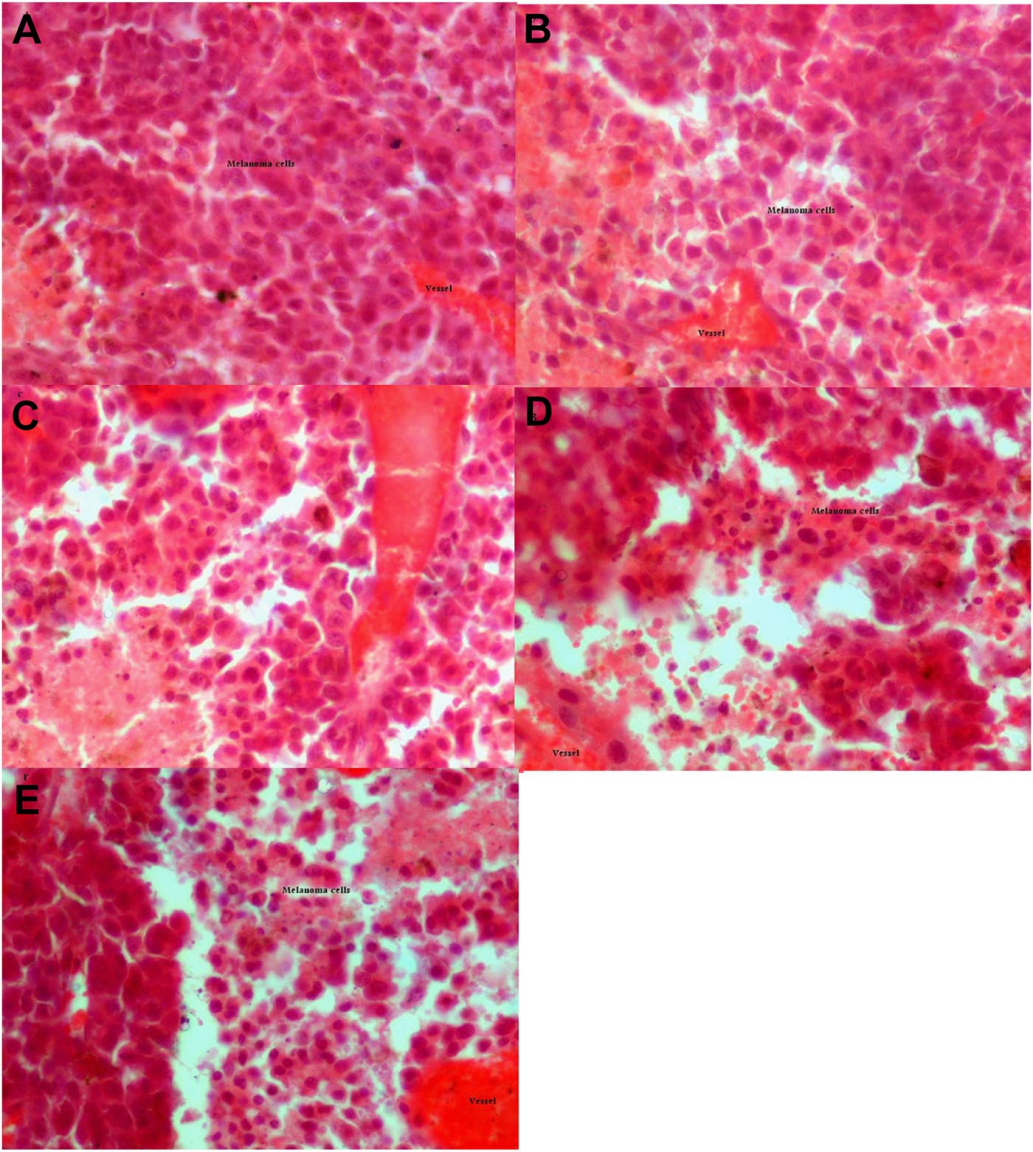
The results of tumor H&E staining have been shown in (**A**) Control group, (**B**) Chemotherapy drug, (**C**) Indirect plasma, (**D**) Direct plasma, (**E**) Combination of indirect plasma and chemotherapy drug. The tissue sections were prepared from a 2–3 mm depth. The scale bar of images is 500 μm.The scale bar of images is 625 μm. (P-values < 0.05 (*), P-values < 0.01 (**) and P-values < 0.001 (***)).

H&E staining was performed to investigate the differences between the control and the treatment groups. The histological results showed that the density of the cells showed significant differences between the control and the treatment groups. The results showed that the number of cells in the treatment groups are less than the control group (Fig. 7), and there were no signs of burning in the observed tissues in treatment groups.

### TUNEL assay

**Figure 8:**
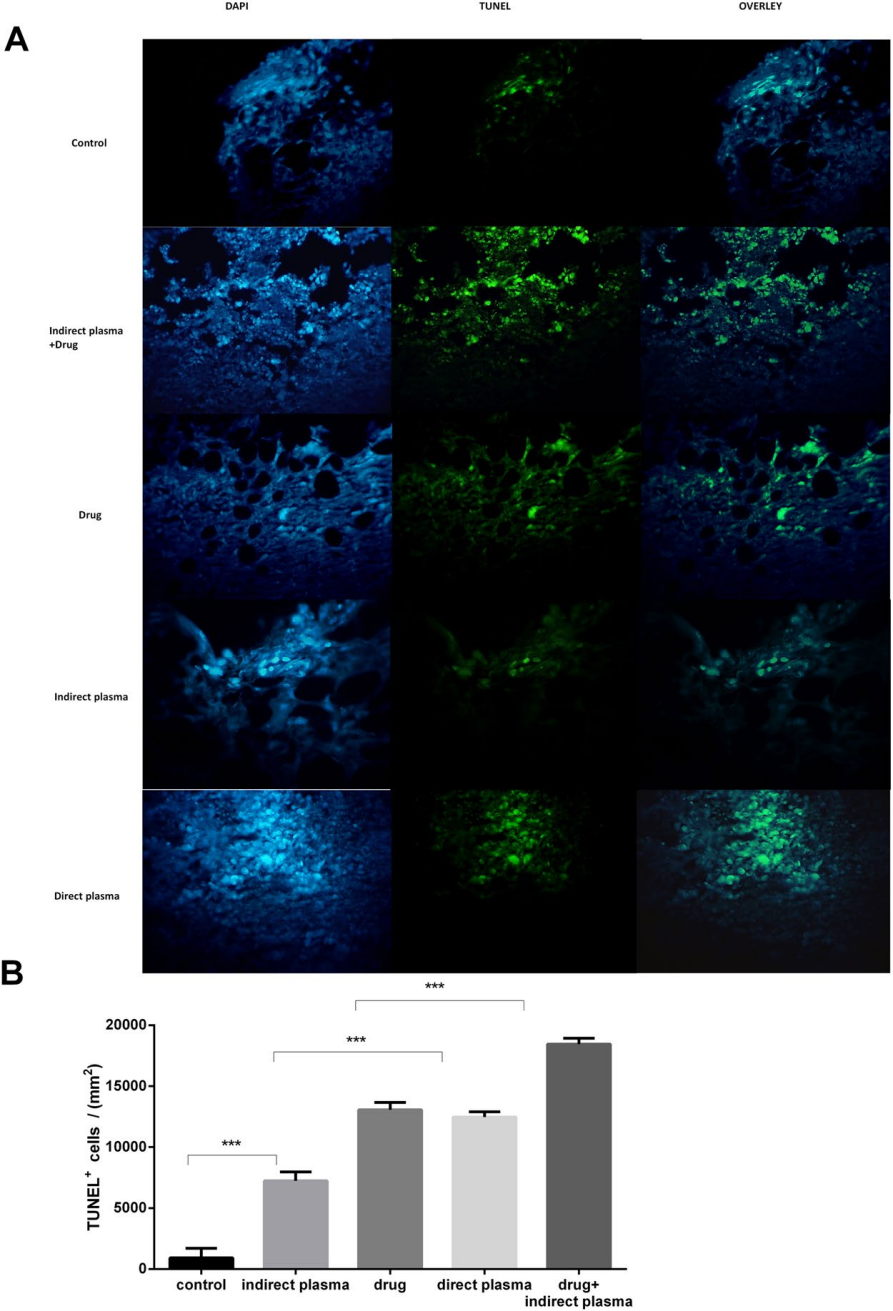
(**A**) Analysis of apoptosis and DNA damage by using TUNEL assay. These nuclei were staining with TUNEL kit after drug injection and plasma exposure. TUNEL and DAPI images were merged and produced overlay images. (**B**) Average of TUNEL positive cells (count)/10^3^ mm^2^ in untreated, combination of indirect plasma and chemotherapy drug, drug, indirect plasma and direct plasma tumor tissues. The scale bar of images is 625 μm. (P-values < 0.05 (*), P-values < 0.01 (**) and P-values < 0.001 (***)).

Nuclei were stained by DAPI, apoptotic cells were shown as TUNEL positive reaction and nuclei were merged by positive reaction cells in Fig. 8. According to the results of the TUNEL test, shown in Fig. 8, average of TUNEL positive cells (count)/10^3^ mm^2^, in the combined, direct plasma, chemotherapy drug, indirect plasma, and control groups was 18.43, 12.46, 13.06, 7.22, and 0.90, respectively. Apoptotic cells were not noticeably seen in untreated group. These date provided evidence regarding the therapeutic potential of the combined group as an anticancer drug in melanoma cancer cells (P < 0.05) (Fig. 8).

### Western blot result

**Figure 9:**
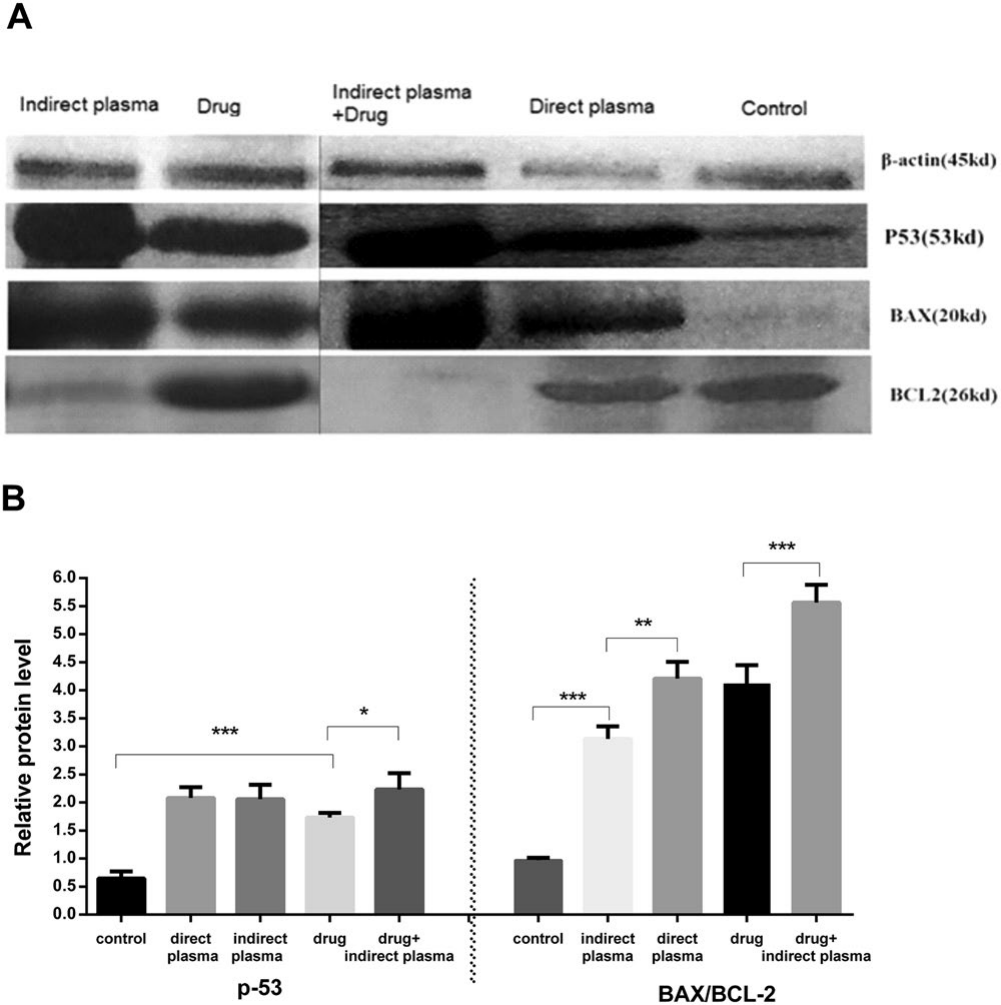
(**A**) The results of western blotting have been shown using antibodies Bax, Bcl–2 and p-53 in treatment and control group. The images of control, direct plasma and indirect plasma + drug groups were plotted on a same gel and the images of drug and indirect plasma groups were plotted on another gel. (**B**) Relative protein levels in 5 different groups. (P-values < 0.05 (*), P-values < 0.01 (**) and P-values < 0.001 (***)).

Figure 9 shows the expression of p-53, Bax and Bcl-2 in melanoma tumor cells after treatment. The Bax protein is impeller of cell death. It has been proposed the relative sensitivity of cancer cells to apoptotic exciter is controlled by the rate of Bax/Bcl-2 and other Bcl-2 family proteins. Direct and indirect plasma, chemotherapy and combinational therapy increased the level of p-53 protein expression and Bax/Bcl-2 ratio. Therefore, this rate plays an important role in the apoptotic of tumor cells after treatment.

### Radiology and CT scan

**Figure 10:**
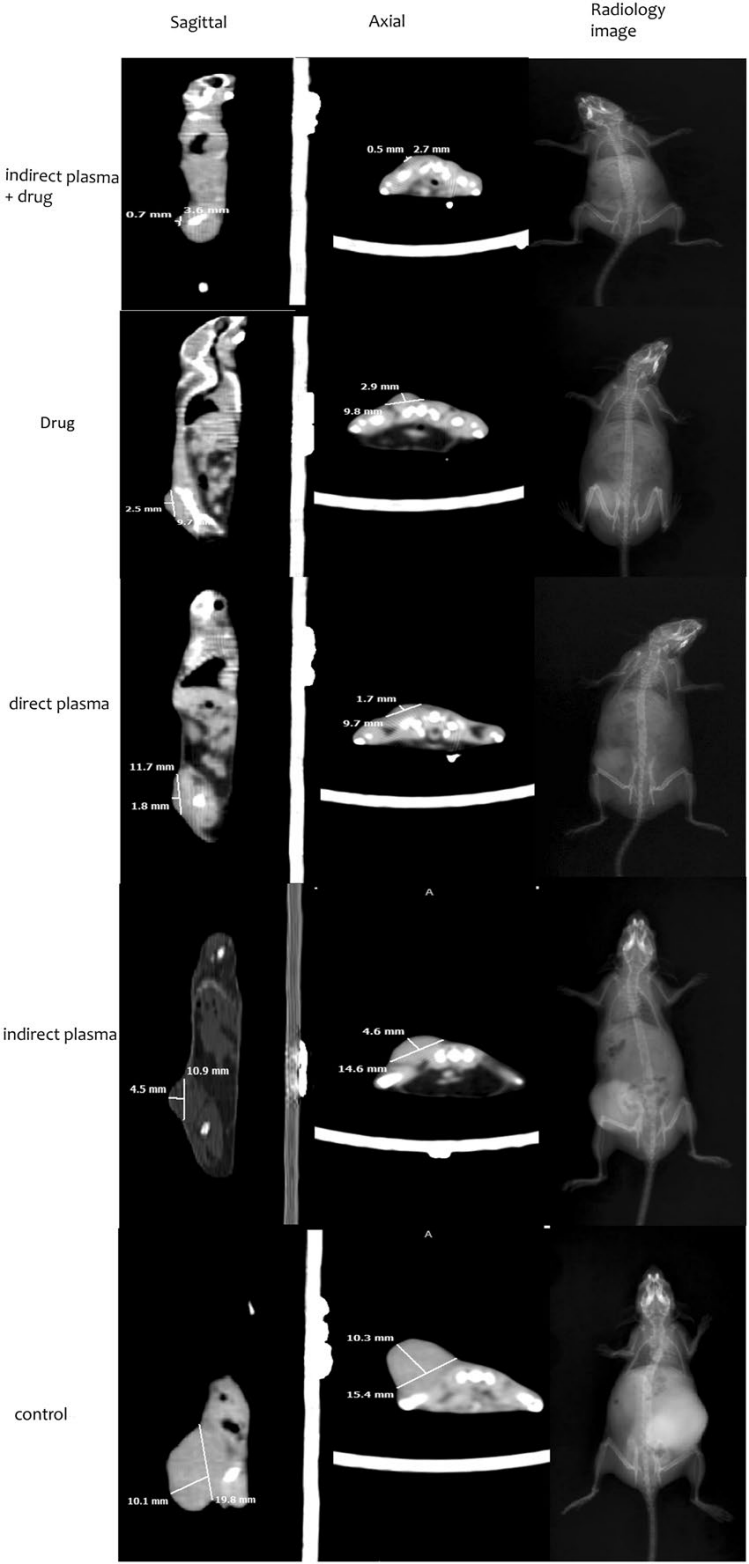
Radiology, sagittal and axial CT scan images of mice with melanoma cancer in all groups after intervention.

After the treatment of the mice tumor, the size of the tumor was evaluated by radiological and CT scan image. As shown in Fig 10 and Table 1, although direct plasma treatment was more effective than indirect treatment, the combined method showed a dramatic tumor size reduction. Radiological images also indicate that no metastatic effects had occurred.

**Table 1:** Shows the length, width, height tumor of mice based on CT scan results.

Group name	Length(mm)	Width(mm)	Height(mm)
1. Control group	15.4	19.8	10.1
2. Indirect plasma group	14.6	10.9	4.5
3. Drug group	9.8	9.7	2.5
4. Direct plasma group	9.7	11.8	1.8
5. Combination of Indirect plasma and chemotherapy group	2.7	3.6	0.7

## Discussion

Cancer treatment by CAP is an idea with diverse pathways, including ROSs and RNSs with discontinuation of cell cycle that induces apoptosis^55^. Apoptosis is a physiological and biological process which is necessary to maintain homeostasis in body and if disrupted, it could lead to pathological conditions and diseases^56^. Documentation shows that CAP is an effective treatment for various cancer cell lines and tumors. CAP treatment increases ROS in the cellular levels and bearing DNA damage to cancer cells while not influencing healthy cells vastly^57^. Also, it is shown that ROS reacts with amino acids which leads to membrane damage and lipid peroxidation stimulation^9,55^. Consequently, ROSs could penetrate cell membrane and cause damage to imposed cells^14^. This procedure also increases G_2_/M by double in cancer cells and creates an oxidative stress that gives rise to s-phase cycle damage^42^. Superficial tumors can be treated by direct exposure and obtain notable results^32,58^. Direct exposure is not a practical when the tumor is inside the patient’s body. Nowadays, scientists are researching a method to transfer plasma inside the body in order to inhibit tumor growth^59^. This study was performed to compare direct and indirect plasma treatment and examine their effectiveness of both *in-vivo* and *in-vitro*. The antitumor mechanism of direct and indirect treatment are mostly similar, although in indirect treatment some species will interact with media prior to injection^33^. Cell cultured medium facilitates active species from gas phase to a dissolvable ones in liquid and could transfers these species to interact with cells and their membrane^60^. It should be mentioned that since lifetime of some species such as OH is short, they will be recombined prior to injection^61,62^.

Results in this study indicate that direct CAP treatment is more convincing than indirect one. This could be due to different factors such as the surface of treatment, reactions of plasma reactive species such as OH, H_2_O_2_, NO, O3, etc. with environmental compounds that the plasma collides with and long and short lifetime of reactive species produced by cold plasma. The interaction of cold plasma with cell cultured medium and skin surface is still unknown due to its complex composition of the medium and different layers of the skin.

Although indirect treatment was not as effective as direct method, it has relatively less toxicity on treated cells. So, this observation indicates that indirect method could be used vastly inside the boy with much less side effects^41^. Therefore, pursuing a combined method of indirect plasma with a conventional therapy such as chemotherapy could be more practical because of increased antitumor effects yet reducing the drug dosage and side effects. Results showed that the combination of the indirect plasma exposure with chemotherapy induced a significant reduction in tumor volume^63^. Not only no distractive interference of indirect plasma and chemotherapy was observed but also this method was far more effective than any other methods. In this procedure negative growth of tumor size at *in-vivo* study was observed. These findings are consistent with the results of previous studies which showed that combining a chemotherapy drug such as Temozolomide with CAP had a much stronger impact than each one alone^64^.

In melanoma cells treated with CAP, receivers of tumor’s necrosis factor that are based on apoptosis pathways are activated by increasing intracellular ROSs. Most observed apoptosis paths in cancer cells which are being treated with plasma are based on mitochondrion paths that have been commenced by DNA and mitochondrial damage^65,66^. P-53 phosphorylation that activates pro-apoptotic factors like Bax, is an essential step of cell cycle stopping paths which is necessary to start apoptosis paths based on mitochondria^67,68^. The results showed that the plasma induces apoptosis in the tumor cells by activating the p-53 and Bax/Bcl-2 proteins. We realized that apoptosis in chemotherapy and indirect treatment combination is %66.41, by flow cytometry assay. TUNEL assay indicated similar results *in-vivo*. This also proves that the combined method is as effective as mentioned before.

As a confirmation, the results of radiology and CT scan show the reduction in tumor size in treated group compared to control group. Also, according to the results it can be understood that cold plasma is an option to inhibit metastasis of malignant cancer.

The difference in apoptosis rate with other studies is due to the experiment conditions such as concentration of the active species^69^, FBS concentration in cell cultured medium for cell growth rate^70^, and the number of cells per unit volume of the medium^41^.

One of the issues raised in the plasma treatment is the thermal damage implied to tissue^71^. It can be said that the effect of ultraviolet radiation, heat and magnetic fields are negligible on the cells^49,72^. By measuring cell cultured medium temperature by an Infra-Red camera, it was observed that cell cultured medium temperature after 6 minutes of plasma exposure increased to 36.2 °*c*. Considering that standard incubator temperature is 37 °*c*, it could be concluded that plasma treatment has no thermal effects on cancer cells. Also, mouse skin temperature was evaluated *in-vivo* experiment and temperature increase was reported about 1 ± 0.3 °*c*, showing that this change in temperature cannot inflict any thermal damages. In this regard, the result of H&E staining after plasma exposure proves that cold plasma doesn’t cause any thermal damages^73^.

Furthermore, since ultraviolet photons and thermal effects are negligible factors, the observed cellular response is mostly due to the impact of different species generated by cold plasma^72,74^. Even after disappearing the tumor with CAP treatment there were no signs of skin damage. Since ROS levels in cancer cells are higher and antioxidant levels are lower than those in normal cells, they reach the threshold of apoptosis rapidly when plasma is radiated on the tumor^18,45^. Plasma is an adjustable source of active species; therefore, cellular response to plasma exposure is due to the production and composition of these species^48^.

In summary, this study showed that although the direct plasma treatment effectiveness is more than indirect treatment, the plasma activated medium showed great potential for tumors located inside the body or when plasma device is not available.

The present study revealed that the combination of the plasma activated medium with other conventional cancer treatment methods such as chemotherapy has more antitumor effects than direct plasma and chemotherapy alone; it also reduces the side effects and the drug dosage as well.

## Material and Methods

### Ethics statement

In this study 8–10 week– C57 female mice were purchased, and kept in laboratory animal house of Shahid Beheshti University of medical science in clean cages with free access to mouse food and water to reach the 18–20 gr weight. Laboratory animal house temperature was about 24 °*c* and their lighting program was controlled 12 hours of light and 12 hours of darkness. Mice at the time of surgery were calm with anesthesia injection. All mice conditions were equal at all times and were in accordance with the animal ethical statement of Shahid Beheshti University of medical science.

### Plasma setup and characterization

The plasma jet device consists of a copper tube as a central electrode, the copper ring as a ground electrode and the Acrylonitrile Butadiene as a dielectric barrier. The copper tube was connected to the high voltage power supply and the copper ring was connected to the ground. In this setup a 25 kHz AC power supply with the voltage of 5 kV was used. Helium gas was chosen as the carrier gas with a flow rate of 4slm. The range of plasma plume length was (22 ± 1) mm and the distance between the nozzle and floor plate or the skin surface of the mice was 15 mm. To determine the characteristics of plasma, two types of spectrometers Avaspec-3648-USB_2_ with a wave length of (200–1100) nm and with (0.6–0.7) resolution and Ava spec ULs 3648 usB_2_ with a wavelength of (280–440) nm and with (0.09–0.1) nm resolution were used to identify the type and intensity of species in plasma. Emission spectrum of plasma was collected by using an optical fiber, which was placed vertically near the plasma plume, and spectrum obtained from plasma was analyzed by Ava soft 7.5.3 software.

### Study methods and plasma exposure

**Figure 11:**
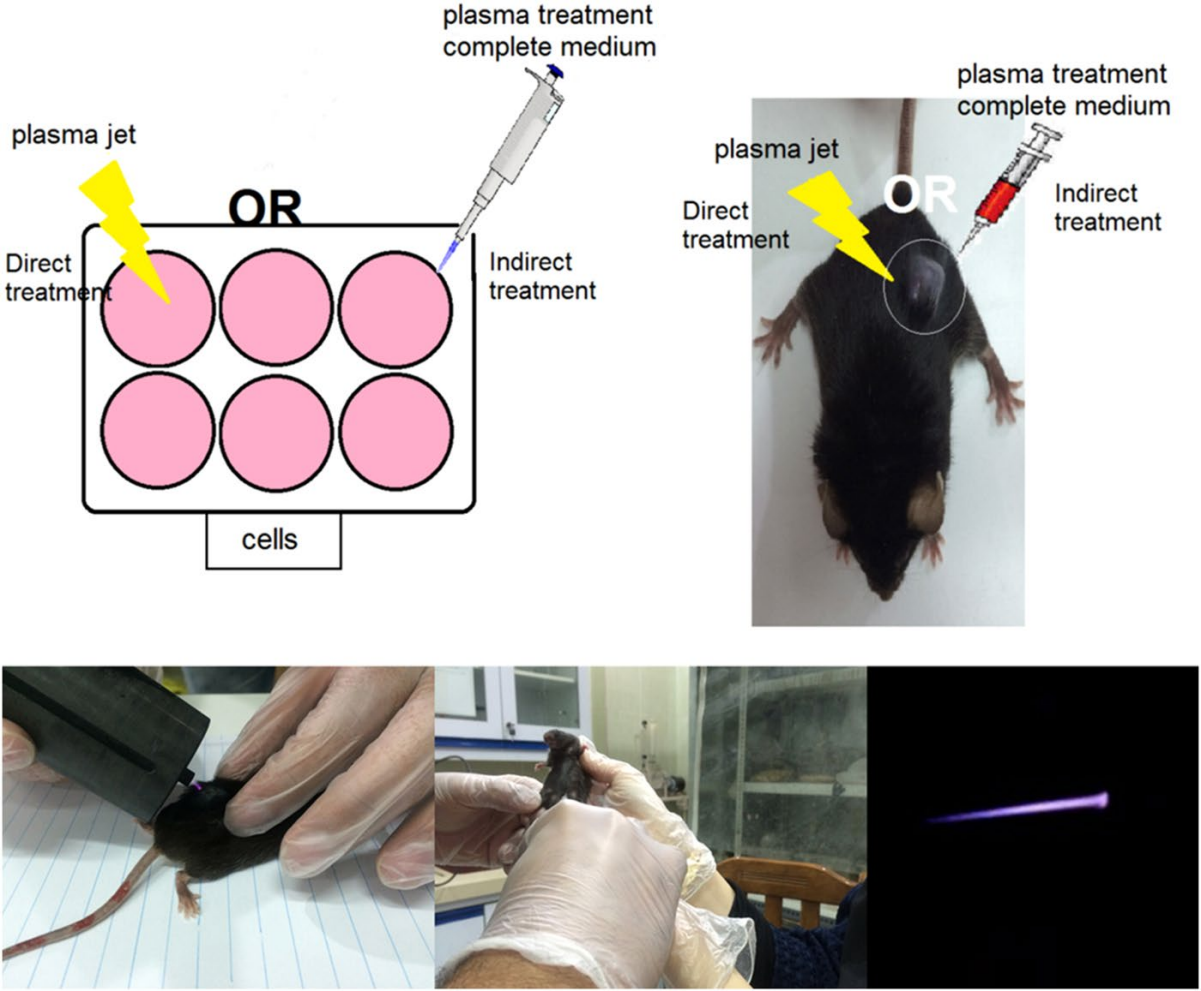
Schematic image of the experiment setup and various methods of treatment.

*In-vivo* and *in-vitro* studies were conducted on treatment of the mouse metastatic melanoma cancer. Two types of treatments by using CAP jet, which have been used for cancer treatment as shown in Fig. 11, are direct and indirect techniques.

In the direct method, CAP was imposed directly to the cells and the mouse tumor. In the indirect method, 1 mL of cell culture medium treated by CAP with different doses in a 6-well plate. Immediately after, certain volume of CAP activated medium was used for cancer cells treatment *in-vivo* and *in-vitro* studies. There exists a drastic distinction between plasma plume delivered in ambient air and plasma plume delivered on the mouse, as evidenced by the photographs in Fig. 11. In addition, target is very influent on the plasma features^53,75,76^.

### Cell cultured and plasma treatment

The murine metastatic melanoma B_16_F_10_ cancer cells (Pasteur institute, IR) were cultured in a complete medium containing Dulbecco’s modified Eagles medium, DMEM (Gibco Co, USA), 15% (v/v) fetal bovine serum (FBS) (Gibco Co,USA), 2mM L-Glutamine, 1% (v/v) penicillin and streptomycin solution (sigma- Aldrich, USA) as an antibiotics. The cells were incubated at 37 °*c* under 5% co_2_ in the Shahid Beheshti University Labs.

Cyclophosphamide (Sigma-Aldrich, USA) which can act as an apoptosis-inducing agent was used at the concentration of 500 μg/ml as a conventional chemotherapeutic drug^77^.

### MTT assay

MTT assay was performed to determine the cytotoxicity of direct and indirect plasma treatments and to find the optimum doses. B_16_F_10_ melanoma cells were cultured at density of 4 × 10^4^ cells per well in the complete medium. Cells’ conditioned medium was removed after 24 hours and CAP irradiated in the direct and indirect methods as follows.

In the direct method, a complete medium was added to the cells and then they were treated by CAP for different dosages of 0, 2, 4 and 6 minutes.

In the indirect method, 1 mL a complete medium was transferred to a 6-well plate, and was treated by CAP for 0, 2, 4, and 6 minutes. Then the plasma activated medium was added to each of the 6-well plates.

Direct and indirect treatments were compared and the optimum dosage of plasma was determined, second comparison was conducted to investigate the effect of combination of indirect plasma and common therapies such as chemical drug. In the direct treatment, cell supernatant culture medium was replaced with 1.5 ml of complete medium and then CAP was imposed for 6 minutes.

In indirect treatment, 1.5 ml of completed medium was treated by CAP in 6-well plate for 6 minutes; afterwards the complete activated medium was transferred on the cells. In chemotherapy group, cells received 500 μg/ml of cyclophosphamide and in a combination of chemotherapy and indirect CAP treatment group cells received 500 μg/ml of cyclophosphamide and also received 1.5 ml plasma activated medium.

### Flow cytometry

Flow cytometry analysis was done to measure cell apoptosis. 4 × 10^4^ B_16_F_10_ melanoma cells were cultured in 5 different groups with six repeats. According to the results of MTT assay, flow cytometry tests were done 48 hours post treatment. After treatment, the cells were trypsinized and were washed twice with Phosphate-Buffered Saline (PBS), centrifuged at 2000 revolutions per minutes (RPM) for 5 minutes and added 500 μL of the binding buffer. When cells were separated from each other, cell suspensions were prepared and were stained by flow cytometry protocol.

### Animal study

1 × 10^6^ B_16_F_10_ melanoma cells in DMEM were injected subcutaneously to 36 female C57BL/6 mice aged 8–10 weeks (purchased from Pasteur, IR). After a week, when tumor size reached to (5–6) mm, the experiment was separated into two categories. First a comparison was made between direct and in direct plasma treatments and afterwards the combined group with others. Combination of chemotherapy and plasma treatment was implemented to investigate possible interferences. In direct CAP treatment group, CAP was imposed directly to surface of the tumor for 6 minutes and in indirect CAP treatment group, mice received 400 μL of activated medium which was activated for 6 minutes by plasma exposure. Also, the same volume of DMEM medium that has not been exposed by plasma was injected to the other groups to investigate DMEM possible side effects and qualities of the therapy. In the drug treatment group, each mouse received 130 $\frac{mg}{kg}$ cyclophosphamide, and in the combined group, each mouse received administered i.p cyclophosphamide at the dose of 130 $\frac{mg}{kg}$ and were treated by 400 μL activated medium^78^. Treatment was performed every day at the same time and for 25 days. Tumor volume was calculated using the formula V = 0.52 × (X^2^Y) for every 5 day and for all groups. For tumor extraction, mice were anesthetized with ketamine (100 $\frac{mg}{kg}$) and xylazine (10 $\frac{mg}{kg}$) (Alpha, Co.IR) and fixed in 10% paraformaldehyde for 1 month.

### Hematoxylin and Eosin staining

Histological evaluation was performed at 25 days after direct and indirect treatments. Every tumor was removed and tissue samples were fixed in 10% formalin for 1 month. Then the tissue samples were embedded in paraffin blocks and serial sections (10 μm thick) were made using a microtome. For the microscopic descriptive analysis of each group, sections were stained with H&E in order to estimate the density of the cells of the tissue samples.

### TUNEL assay

DNA fragmentation refers to apoptosis markers. Terminal deoxynucleotidyl transferase dUTP nick and labeling (TUNEL) is a method to detect DNA fragmentations and was used to evaluate the induced apoptotic effects of plasma therapies. This test was performed using situ Cell Death Detection kit (fluorescence, Roche, CH). After treatment, the xenograft B_16_F_10_ tumors growing in mice were harvested at necropsy, fixed and embedded in paraffin and mounted on glass slides, deparaffinized with xylene, rehydrated in a piecemeal series of ethanol, washed in H_2_O and subjected to TUNEL staining. Assay was performed according to the TUNEL protocols. The images of all groups obtained by TUNEL were processed by image j software and then the diagram of TUNEL positive cells/10^3^ mm^2^ were indicated for all groups^79^.

### Western blotting

After evaluating the effects of direct, indirect, and combined therapy on proliferation and apoptosis, several proteins activation such as p53, Bax, Bcl-2 and β-Actin were investigated by western blotting. Half of the tumors were extracted from the animals, to determine the protein level in tissue of all groups. To perform this analysis, tissues were washed twice with PBS, then squished and combined with buffer and centrifuged for 15 minutes. The protein concentration was determined by applying the Bradford method. The proteins were dissevered by electrophoresis with sodium dodecyl sulfate-polyacrylamide gel (SDS-PAGE) and then transferred onto polyvinylidene difluoride (PVDF). Finally, they were investigated with primary antibody including rabbit polyclonal anti-Bax (1:200), mouse polyclonal anti-Bcl2 (1:200), rabbit polyclonal anti-P53 proteins (1:200) (Santa Cruz Biotechnology, USA) and secondary antibodies conjugated with horse radish peroxidase (HRP) (Cell Signaling Technology, USA).the β-Actin generation signal was used as an internal control.

### CT scan and Radiology

To perform radiology examinations and CT scan in controlled and treated groups, mice were transferred to the CT scan and radiology of Taleghani clinic. Radiation in CT scan was performed at zoom of 1.08, axial and sagittal cutting with 2 mm thickness, 10 mA current and 120 kV voltage. Radiology examinations were performed at tube current-time of 220 mAs and voltage of 80 kV.

### Statistical analysis

Results were expressed as mean ± standard deviation (mean ±SD) and calculated by Spss Software. One way-ANOVA was used for comparing the groups with each other. In addition, student t-test was employed to compare the means of each group in tumor volume.

### Ethical Considerations

The proposal of study was approved by the Ethics Committee, deputy of research, Shahid Beheshti University of Medical Sciences, Tehran.

## Electronic supplementary material

Supplementary Dataset

## References

[1] Weltmann K, von Woedtke T (2016). Plasma medicine—current state of research and medical application. Plasma Physics and Controlled Fusion.

[2] Suschek CV, Opländer C (2016). The application of cold atmospheric plasma in medicine: the potential role of nitric oxide in plasma-induced effects. Clinical Plasma Medicine.

[3] Heinlin J (2010). Plasma medicine: possible applications in dermatology. JDDG: Journal der Deutschen Dermatologischen Gesellschaft.

[4] Nezhat C, Kho KA, Morozov V (2009). Use of neutral argon plasma in the laparoscopic treatment of endometriosis. JSLS: Journal of the Society of Laparoendoscopic Surgeons.

[5] Robotis J, Sechopoulos P, Rokkas T (2003). Argon plasma coagulation: clinical applications in gastroenterology. Annals of Gastroenterology.

[6] Mai-Prochnow A, Murphy AB, McLean KM, Kong MG, Ostrikov KK (2014). Atmospheric pressure plasmas: infection control and bacterial responses. International journal of antimicrobial agents.

[7] Arora V, Nikhil V, Suri N, Arora P (2014). Cold atmospheric plasma (CAP) in dentistry. Dentistry.

[8] Stoffels E, Roks AJ, Deelman LE (2008). Delayed effects of cold atmospheric plasma on vascular cells. Plasma Processes and Polymers.

[9] Volotskova, O., Hawley, T., Stepp, M. A. & Keidar, M. Cold Atmospheric Plasma as an alternative therapy for cancer therapies. *Bulletin of the American Physical Society* **57** (2012).

[10] Chen, F. F. *Introduction to plasma physics*. (Springer Science & Business Media, 2012).

[11] Biberman, L. M., Vorobʹev, V. S. & Iakubov, I. Kinetics of nonequilibrium low-temperature plasma. *Moscow Izdatel Nauka* (1982).

[12] Harper JD (2008). Low-temperature plasma probe for ambient desorption ionization. Analytical chemistry.

[13] Tachibana K (2006). Current status of microplasma research. IEEJ Transactions on Electrical and Electronic Engineering.

[14] Kim SJ, Chung T (2016). Cold atmospheric plasma jet-generated RONS and their selective effects on normal and carcinoma cells. Scientific reports.

[15] Kim SJ, Chung T, Bae S, Leem S (2010). Induction of apoptosis in human breast cancer cells by a pulsed atmospheric pressure plasma jet. Applied Physics Letters.

[16] Kalghatgi S (2011). Effects of non-thermal plasma on mammalian cells. PloS one.

[17] Liebmann J (2011). Biological effects of nitric oxide generated by an atmospheric pressure gas-plasma on human skin cells. Nitric Oxide.

[18] Thiyagarajan M, Anderson H, Gonzales XF (2014). Induction of apoptosis in human myeloid leukemia cells by remote exposure of resistive barrier cold plasma. Biotechnology and bioengineering.

[19] Zhao S (2013). Atmospheric pressure room temperature plasma jets facilitate oxidative and nitrative stress and lead to endoplasmic reticulum stress dependent apoptosis in HepG2 cells. PloS one.

[20] Graves DB (2014). Reactive species from cold atmospheric plasma: implications for cancer therapy. Plasma Processes and Polymers.

[21] Pavelescu L (2015). On reactive oxygen species measurement in living systems. Journal of medicine and life.

[22] Iza F (2008). Microplasmas: sources, particle kinetics, and biomedical applications. Plasma Processes and Polymers.

[23] Cairns RA, Harris IS, Mak TW (2011). Regulation of cancer cell metabolism. Nature Reviews Cancer.

[24] Volotskova, O., Hawley, T. S., Stepp, M. A. & Keidar, M. Targeting the cancer cell cycle by cold atmospheric plasma. *Scientific reports* **2** (2012). 10.1038/srep00636 PMC3434394 22957140

[25] Mirpour S (2016). Utilizing the micron sized non-thermal atmospheric pressure plasma inside the animal body for the tumor treatment application. Scientific reports.

[26] Partecke LI (2012). Tissue tolerable plasma (TTP) induces apoptosis in pancreatic cancer cells *in vitro* and *in vivo*. BMC cancer.

[27] Hoffmann C, Berganza C, Zhang J (2013). Cold Atmospheric Plasma: methods of production and application in dentistry and oncology. Medical gas research.

[28] Vandamme M (2010). Antitumor effect of plasma treatment on U87 glioma xenografts: preliminary results. Plasma processes and polymers.

[29] Schlegel J, Köritzer J, Boxhammer V (2013). Plasma in cancer treatment. Clinical Plasma Medicine.

[30] Keidar M (2011). Cold plasma selectivity and the possibility of a paradigm shift in cancer therapy. British journal of cancer.

[31] Dobrynin D, Fridman G, Friedman G, Fridman A (2009). Physical and biological mechanisms of direct plasma interaction with living tissue. New Journal of Physics.

[32] Gay-Mimbrera J (2016). Clinical and Biological Principles of Cold Atmospheric Plasma Application in Skin Cancer. Advances in therapy.

[33] Utsumi F (2013). Effect of indirect nonequilibrium atmospheric pressure plasma on anti-proliferative activity against chronic chemo-resistant ovarian cancer cells *in vitro* and *in vivo*. PloS one.

[34] Bourdon A (2016). Numerical and experimental study of the dynamics of a μs helium plasma gun discharge with various amounts of N2 admixture. Plasma Sources Science and Technology.

[35] Olszewski P, Wagenaars E, McKay K, Bradley J, Walsh J (2014). Measurement and control of the streamer head electric field in an atmospheric-pressure dielectric barrier plasma jet. Plasma Sources Science and Technology.

[36] Lunov O (2017). Chemically different non-thermal plasmas target distinct cell death pathways. Scientific Reports.

[37] Fridman G (2007). Floating electrode dielectric barrier discharge plasma in air promoting apoptotic behavior in melanoma skin cancer cell lines. Plasma Chemistry and Plasma Processing.

[38] Robert E (2013). Perspectives of endoscopic plasma applications. Clinical Plasma Medicine.

[39] Sohbatzadeh F, Omran AV (2014). The effect of voltage waveform and tube diameter on transporting cold plasma strings through a flexible dielectric tube. Physics of Plasmas.

[40] Kim JY (2011). Single‐cell‐level microplasma cancer therapy. Small.

[41] Yan, D. *et al*. Principles of using cold atmospheric plasma stimulated media for cancer treatment. *Scientific reports* **5** (2015). 10.1038/srep18339 PMC4683589 26677750

[42] Yan, D. *et al*. Stabilizing the cold plasma-stimulated medium by regulating medium’s composition. *Scientific reports* **6** (2016). 10.1038/srep26016 PMC4865954 27172875

[43] Mohades S, Laroussi M, Sears J, Barekzi N, Razavi H (2015). Evaluation of the effects of a plasma activated medium on cancer cells. Physics of Plasmas.

[44] Tanaka, H. *et al*. Cell survival and proliferation signaling pathways are downregulated by plasma-activated medium in glioblastoma brain tumor cells. *Plasma Medicine* **2** (2012).

[45] Judée F (2016). Short and long time effects of low temperature Plasma Activated Media on 3D multicellular tumor spheroids. Scientific reports.

[46] Cheng X (2014). The effect of tuning cold plasma composition on glioblastoma cell viability. PloS one.

[47] Cross CE (1987). Oxygen radicals and human disease. Annals of internal medicine.

[48] Graves DB (2012). The emerging role of reactive oxygen and nitrogen species in redox biology and some implications for plasma applications to medicine and biology. Journal of Physics D: Applied Physics.

[49] Yan D, Sherman JH, Keidar M (2017). Cold atmospheric plasma, a novel promising anti-cancer treatment modality. Oncotarget.

[50] Ja Kim S, Min Joh H, Chung T (2013). Production of intracellular reactive oxygen species and change of cell viability induced by atmospheric pressure plasma in normal and cancer cells. Applied Physics Letters.

[51] Cheng X (2014). Synergistic effect of gold nanoparticles and cold plasma on glioblastoma cancer therapy. Journal of Physics D: Applied Physics.

[52] Akhlaghi M (2015). On the design and characterization of a new cold atmospheric pressure plasma jet and its applications on cancer cells treatment. Biointerphases.

[53] Yan D (2017). The strong cell-based hydrogen peroxide generation triggered by cold atmospheric plasma. Scientific reports.

[54] Yan D, Nourmohammadi N, Talbot A, Sherman JH, Keidar M (2016). The strong anti-glioblastoma capacity of the plasma-stimulated lysine-rich medium. Journal of Physics D: Applied Physics.

[55] Babington P (2015). Use of cold atmospheric plasma in the treatment of cancer. Biointerphases.

[56] Singh N (2007). Apoptosis in health and disease and modulation of apoptosis for therapy: an overview. Indian Journal of Clinical Biochemistry.

[57] Georgescu N, Lupu AR (2010). Tumoral and normal cells treatment with high-voltage pulsed cold atmospheric plasma jets. IEEE Transactions on Plasma Science.

[58] Kajiyama H (2017). Future perspective of strategic non-thermal plasma therapy for cancer treatment. Journal of clinical biochemistry and nutrition.

[59] Binenbaum Y (2017). Cold Atmospheric Plasma, Created at the Tip of an Elongated Flexible Capillary Using Low Electric Current, Can Slow the Progression of Melanoma. PloS one.

[60] Tanaka H (2015). Cancer therapy using non-thermal atmospheric pressure plasma with ultra-high electron density. Physics of Plasmas.

[61] Schmitt F-J (2014). Reactive oxygen species: re-evaluation of generation, monitoring and role in stress-signaling in phototrophic organisms. Biochimica et Biophysica Acta (BBA)-Bioenergetics.

[62] Attri, P. *et al*. Generation mechanism of hydroxyl radical species and its lifetime prediction during the plasma-initiated ultraviolet (UV) photolysis. *Scientific reports* **5** (2015). 10.1038/srep09332 PMC4367430 25790968

[63] Brullé L (2012). Effects of a non thermal plasma treatment alone or in combination with gemcitabine in a MIA PaCa2-luc orthotopic pancreatic carcinoma model. PloS one.

[64] Köritzer J (2013). Restoration of sensitivity in chemo—resistant glioma cells by cold atmospheric plasma. PloS one.

[65] Ishaq M (2014). Atmospheric gas plasma–induced ROS production activates TNF-ASK1 pathway for the induction of melanoma cancer cell apoptosis. Molecular biology of the cell.

[66] Kim C (2014). Nonthermal plasma induces head and neck cancer cell death: the potential involvement of mitogen-activated protein kinase-dependent mitochondrial reactive oxygen species. Cell death & disease.

[67] Kastan MB (2008). DNA damage responses: mechanisms and roles in human disease. Molecular Cancer Research.

[68] Joerger AC, Fersht AR (2008). Structural biology of the tumor suppressor p53. Annu. Rev. Biochem..

[69] Yan X (2012). Plasma‐induced death of HepG2 cancer cells: intracellular effects of reactive species. Plasma Processes and Polymers.

[70] Ryan JM (1979). Effect of different fetal bovine serum concentrations on the replicative life span of cultured chick cells. In Vitro Cellular & Developmental Biology-Plant.

[71] Brun P (2012). Disinfection of ocular cells and tissues by atmospheric-pressure cold plasma. PloS one.

[72] Mashayekh S, Rajaee H, Akhlaghi M, Shokri B, Hassan ZM (2015). Atmospheric-pressure plasma jet characterization and applications on melanoma cancer treatment (B/16-F10). Physics of Plasmas.

[73] Kos S (2017). Safety aspects of atmospheric pressure helium plasma jet operation on skin: *In vivo* study on mouse skin. PloS one.

[74] Korachi M, Aslan N (2013). Low temperature atmospheric plasma for microbial decontamination. Microbial pathogens and strategies for combating them: science, technology and education.

[75] Yamada H (2016). Spectroscopy of reactive species produced by low-energy atmospheric-pressure plasma on conductive target material surface. Journal of Physics D: Applied Physics.

[76] Darny T (2017). Analysis of conductive target influence in plasma jet experiments through helium metastable and electric field measurements. Plasma Sources Science and Technology.

[77] Kugawa F, Ueno A, Kawasaki M, Aoki M (2004). Evaluation of cell death caused by CDF (cyclophosphamide, doxorubicin, 5-fluorouracil) multi-drug administration in the human breast cancer cell line MCF-7. Biological and Pharmaceutical Bulletin.

[78] Huyan X-H, Lin Y-P, Gao T, Chen R-Y, Fan Y-M (2011). Immunosuppressive effect of cyclophosphamide on white blood cells and lymphocyte subpopulations from peripheral blood of Balb/c mice. International immunopharmacology.

[79] Maidana DE (2015). A Novel ImageJ Macro for Automated Cell Death Quantitation in the RetinaA Novel ImageJ Macro for Cell Death Quantitation. Investigative ophthalmology & visual science.

